# ﻿10 years of advancing diversity within the Mycological Society of America

**DOI:** 10.3897/imafungus.16.155609

**Published:** 2025-05-28

**Authors:** Mycological Society of America Diversity, Equity and Inclusion Committee

**Affiliations:** 1 Mycological Society of America DEI Committee, Windsor, WI, USA Mycological Society of America DEI Committee Windsor, WI United States of America

**Keywords:** DEI, equity, fungal biology, inclusion, STEM

## Abstract

Like most Science, Technology, Engineering, and Mathematics (STEM) fields, mycology has been dominated by white male scientists, while representation of marginalised groups (*e.g.*, women, persons from different racial and ethnic groups, persons with disabilities, and members of the LGBTQ+ community) remains low. Here, we discuss diversity, equity, and inclusion in mycology and report on the last decade’s efforts to improve diversity in this field. We highlight changes and initiatives implemented by the Mycological Society of America and assess their success. We conclude by making recommendations for the next steps required to achieve non-discrimination and balanced representation in mycology.

## ﻿Introduction

Diverse scientific visions enrich communities and lead to novel ideas for research, education, and outreach. Specifically, workforce diversity, including different backgrounds, experiences, and points of view, can be leveraged to catalyse innovation and creativity, thereby contributing to a vigorous Science, Technology, Engineering, and Mathematics (STEM) enterprise (National Science Foundation 2023). Even though achieving diversity in STEM is a United States of America national priority, STEM fields in the country are white- and male-dominated, with several groups being underrepresented in STEM-based postsecondary education and workforce (Colwell et al. 2020; Johnson et al. 2018; Morris and Washington 2018; Ballou et al. 2019; Kraševec 2022; Burt et al. 2023). These include women, persons with disabilities, neurodivergent persons, persons belonging to the LGBTQ+ community, and persons belonging to some racial and ethnic minority groups, namely Hispanic or Latino, Black or African American, and American Indian or Alaska Native (National Science Foundation 2023; Cech and Waidzunas 2021; Morris and Washington 2018; Sanchez et al. 2019). Unfortunately, despite ongoing efforts, diversifying STEM postsecondary education and workforce continues to be a challenge (Segarra et al. 2020; Ballou et al. 2019).

Being at the boundary between higher education and the workforce, scientific professional societies are well-positioned to contribute to diversifying STEM (Sanchez et al. 2019). They encompass scientists with distinct training and career trajectories, from undergraduate students to senior faculty, along with industry, non-profit, and government workers, and play a privileged role in assisting scientists throughout their careers. Societies provide opportunities for obtaining admission to graduate programs and postdoctoral positions, developing project ideas and preparing proposals for funding, publishing and presenting research, establishing collaborations and networks, providing/receiving mentorship, and exploring career opportunities (Potvin et al. 2018). Scientific societies can easily create and implement programs that empower underrepresented scientists, assist with their career progression, and lay the foundation for their persistence and longevity in STEM fields. Moreover, scientific societies serve as the liaison between career-seeking researchers and educational and professional opportunities. They can catalyse broad interest, participation and retention of underrepresented groups in STEM, ultimately having the potential to achieve and maintain diversity in the Nation’s educational systems and future workforces (Morris and Washington 2018). However, broad participation in scientific societies is often not achieved (*e.g.*Branco and Vellinga 2015), rendering the need for purposeful DEI action.

Here, we discuss diversity, equity, and inclusion in mycology and document efforts to improve the state of the field. We specifically focus on the initiatives implemented during the past decade by the Mycological Society of America (MSA), a scientific society based in the United States of America that is dedicated to promoting and advancing mycology. We conclude with a summary of recommendations for the next steps needed to achieve balanced representation in the field of Mycology.

## ﻿Mycological Society of America

The Mycological Society of America (MSA) was founded in 1932 and has the purpose to promote and advance the science of mycology and to foster and encourage research and education in mycology in all its aspects (Mycological Society of America 2022a). As of 2023, there were 956 MSA members, mainly from North America, but also from 39 other countries across the globe. Like many other scientific societies, MSA holds an annual meeting that is a forum for professional networking and the dissemination of research findings.

**Figure 1.**
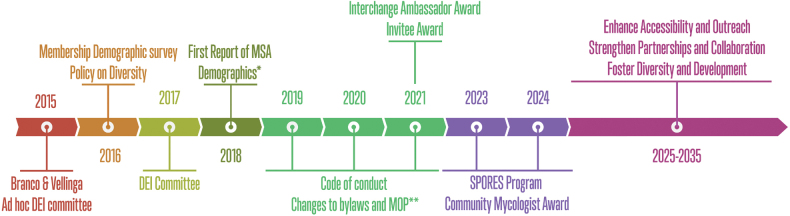
Efforts of the MSA Diversity, Equity, and Inclusion Committee over time, highlighting initiatives to promote and support diverse participation within the society.* Cheeke et al. 2018. **MOP, Manual of Operations.

In 2015, 90 years after the founding of MSA, a study of gender imbalance in mycology rocked the perception of an equitable and welcoming society (Branco and Vellinga 2015). The report compiled gender data for different facets of the MSA, including its membership, officers, and award recipients, as well as the gender proportion of authors who published in the society’s journal *Mycologia* over three different years that spanned decades (2014, 1980, 1946). Branco and Vellinga (2015) reported that women were underrepresented in mycology, particularly within the MSA, making it far from a gender-balanced field and society. Specifically, the authors reported ~60% male membership and much higher proportion of male representation in officer positions and professional awards, for example with 87% male presidents and 92% male Distinguished Mycologists between the beginning of MSA and 2015 and 81% male presidents and 90% male Distinguished Mycologists between 1996 and 2016. The findings were received with surprise by the MSA membership and prompted a call to action. Over the past decade, MSA has actively invested in improving diversity, equity and inclusion (DEI) by implementing a series of initiatives aimed at diversifying both its membership and the field of Mycology (Fig. 1).

### ﻿MSA’s Diversity, Equity and Inclusion Committee

Following the publication of Branco and Vellinga (2015), the MSA created a DEI committee responsible for enhancing awareness and promoting diversity, inclusion, and accessibility in mycology, especially within the society (Mycological Society of America 2022b). In conjunction with the MSA Executive Council, it developed best practices for MSA committee activities that were incorporated into the MSA Constitution and Bylaws and the MSA Manual of Operations (Mycological Society of America 2022a, 2022b). These included specifically stating that “MSA provides an inclusive environment for everyone interested in mycology without regard to gender, gender identity and expression, sexual orientation, disability, physical appearance, race, ethnicity, age, professional status, or religion” and that “all members have the opportunity to participate fully in the society governance, annual meetings, and activities sponsored by the MSA” (Mycological Society of America 2022a).

In 2020, the DEI committee and the MSA Executive Council drafted and implemented a Code of Conduct for MSA along with guidelines for facilitating ‘Safe Mycology’. The latter includes the implementation of the MycoAlly program that trains MSA members to provide support to victims of harassment and report conduct violations that occur at MSA meetings and events, providing a safe, productive, and welcoming environment for all participants. In addition, the DEI committee has organised diversity-oriented events at the annual MSA meetings. These events disseminate the results of MSA diversity surveys and promote discussion on the best approaches for improving diversity. For example, an event entitled ‘Mycology is Diverse’ was held at the 2023 MSA meeting in Flagstaff, Arizona, that included results from the MSA demographics survey, a presentation on the impact of Dr. Lafayette Frederick’s mentorship for six generations of underrepresented minority scientists, and a panel discussion on diversity in mycology. The DEI committee has also developed and implemented a periodic membership diversity survey that allows for the collection of demographic information on MSA members. For example, in 2018 MSA members were predominantly male and white, with 10% identifying as LGBTQ+ and 13% identifying as a person with disabilities (Cheeke et al. 2018). Such information enables the assessment of initiatives meant to improve diversity, equity, and inclusion in the society. MSA was one of the first societies in biology to implement a membership survey, serving as a model for other scientific societies, such as the Society for the Study of Evolution (Rushworth et al. 2021).

More recently, the DEI committee created the MSA Interchange and Invitee Ambassador Awards, was instrumental in changing an award name, and dedicated major efforts to develop and implement SPORES (Society Prioritizes Our Recruitment of Every Scholar), a student mentorship program with the mission to diversify, advance, and promote the retention of future mycologists. The following sections offer context and provide details on these initiatives.

### ﻿MSA Interchange Ambassador Award

To improve the long-term commitment for promoting diversity, equity and inclusion at MSA, the Executive Council and DEI committee established the Interchange Ambassador Award (Fig. 1, Branco et al. 2021). This initiative was developed to disseminate mycology to underrepresented communities through engagement and outreach activities between national scientific societies and organisations, who support DEI in STEM disciplines, and United States of America institutions of higher education designated as Historically Black Colleges and Universities (HBCUs), Hispanic Serving Institutions (HSIs), Tribal Colleges, and other Minority Serving Institutions. So far, there have been three recipients of the MSA Ambassador Interchange Award. They developed several activities within and outside MSA with the overall goal of training students, including the development of the SPORES mentorship program (see details below).

### ﻿MSA Interchange Invitee Award

MSA also implemented the Interchange Invitee Award, which was designed to financially support exceptional scientists (representing any academic level at or beyond the undergraduate stage) from underrepresented groups to attend the MSA annual meeting. Applicants are actively engaged in mycology, interested in joining the MSA (if not a recent member), and willing to present their research at the annual meeting.

### ﻿Code of conduct and changes to MSA Bylaws and manual of operations

The MSA DEI committee was involved in introducing changes to the society’s bylaws and Manual of Operations with the overarching goal of integrating diversity, equity and inclusion principles into the society.

The committee developed ‘Safe Mycology’, a set of rules for making MSA meetings and events inclusive. These involved offering bystander intervention (Myco Ally) training and displaying posters explaining types of harassment at MSA meetings. The committee also compiled a Reporting Protocol for incidents of harassment at MSA events. The harassment policy declares that harassment instances will be dealt with by the executive Vice president or another Council member (Mycological Society of America 2022b). This work culminated in 2020 when the committee finalised the MSA Code of Conduct (https://msafungi.org/msa-code-of-conduct/) for virtual and in-person meetings and other society-sponsored events. The MSA Code of Conduct requires acknowledgement when registering for MSA meetings and events, and when registering as a society member or renewing an MSA membership.

The DEI committee also produced an MSA Statement on Diversity (https://msafungi.org/msa-diversity-statement/) defining the society’s commitment to diversity, inclusion and equity. This statement is meant to be acknowledged, considered, and used as guidance when selecting MSA award recipients. The committee also implemented changes to the MSA Manual of Operations to match the DEI goals of the society. For example, the Manual of Operations now includes a policy to avoid conflict of interest for members of award committees.

To continue to improve the accessibility and inclusivity of MSA annual meetings, the committee actively works with the meeting organisers, providing recommendations for achieving diverse, inclusive, and accommodating meetings. The committee also produced a self-assessment checklist for conference organisers to quantify accessibility and inclusivity performance (Mycological Society of America 2022b).

The DEI committee catalysed other changes, for example the implementation of waivers for SPORES mentee MSA meeting registration fees. In addition, the committee has worked to remove barriers for participation, including proposing changes to MSA membership categories (to better balance fee amount in face of career level income differences). These were an important addition to the 50% reduction in dues for members from low to middle income countries (as defined by the World Bank) already implemented by MSA.

**Figure 2.**
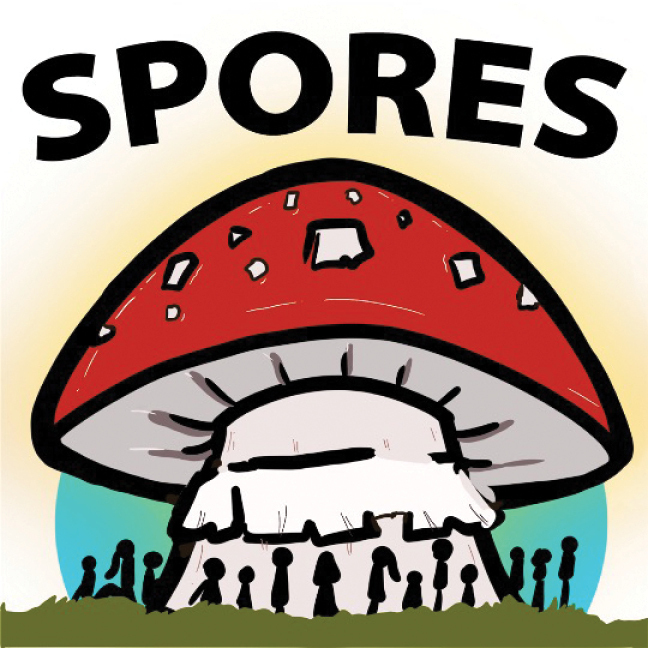
MSA SPORES program logo.

### ﻿SPORES – Society Prioritizes Our Recruitment of Every Scholar

In 2023 the MSA implemented SPORES, a mentorship program to recruit and support undergraduate students from underrepresented demographics at MSA (mentees) and the field of Mycology. Research has shown underrepresented undergraduate students face barriers and that mentoring tailored to undergraduate students not only benefits them but also effectively diversifies STEM fields. For example, minoritized first-year college students enrolled in STEM degrees are highly likely to switch to non-STEM majors or not complete their STEM degree (Tsui 2007), and students at the intersection of marginalised communities are known to have little access to STEM career options (Brown 1995; Griffith 2010). In addition, few undergraduate students can attend science conferences, and even when they do, the experience can be daunting. Underrepresented undergraduate students often find themselves outside networks within scientific societies and lack the skills and/or knowledge to establish connections, which can result in unwelcoming and alienating experiences (Morris and Washington 2018).

The SPORES program mission is to assist and empower underrepresented undergraduate students to attend their first MSA meeting in a welcoming environment. It was strategically designed to pair first-time MSA undergraduate student attendees from underrepresented communities (mentees) with MSA members (mentors), with the aim of diversifying membership and fostering mentee’s academic and professional growth. It provides a supportive environment that prepares students to navigate the social norms of academia and build professional networks, contributing to their retention at MSA and/or mycology and effectively assisting in diversifying STEM fields. SPORES actively contributes to and aligns with MSA’s commitment to inclusivity, equality, and diversity, and attracts undergraduate students from underrepresented and underserved communities at the local, regional, and global levels to become members of the MSA and participate in annual MSA meetings.

**Figure 3.**
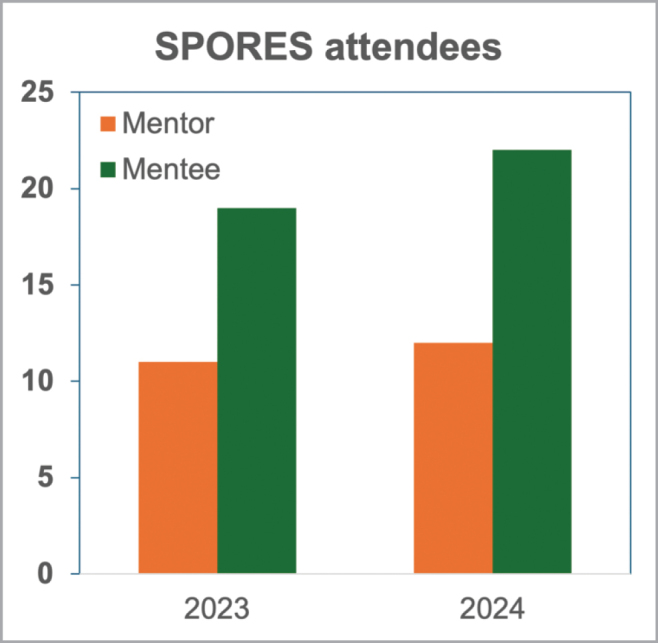
Number of SPORES participants (mentees and mentors) in 2024 and 2023.

MSA successfully implemented SPORES in 2023 and 2024. In its debut year, it hosted 19 mentees (Fig. 3) from diverse backgrounds, representing 18 universities from various regions across the United States and diverse countries worldwide (Canada, México, Hungary, Colombia, and Costa Rica). It also recruited 11 volunteer mentors from six universities at different career stages, including Ph.D. students, postdoctoral researchers, and professors (SPORES Committee 2023). Through support from the MSA, the National Science Foundation (NSF), and generous donors, in 2023 each mentee received one year of MSA membership, conference registration, annual foray fee, and banquet dinner fee. The program was assessed through pre- and post-conference surveys that provided valuable information on the program’s impact and allowed to improve the program. After feedback on the pilot run, it became clear that further funding to support all mentees’ costs in participating in the MSA meeting was a necessity. In 2024, the SPORES program successfully continued. In addition to the 2023 support, SPORES 2024 provided mentees with US$1,500 to cover transportation and lodging expenses, and mentors with US$750 for travel/lodging expenses and the banquet dinner fee. In 2024, SPORES hosted 22 mentees from 20 universities in the United States and Canada, and 12 mentors representing 9 universities and diverse career stages (Fig. 3). In addition, a 2023 SPORES awardee returned to the MSA meeting in 2024 and acted as an advocate for the program and as a liaison between SPORES mentors and the new cohort of mentees. This demonstrates that even in the early stages, the SPORES program is boosting DEI efforts and membership retention.

### ﻿Community Mycologist Award

Along with the initiatives described above, the MSA DEI committee also catalysed the change of an MSA award name. The MSA ‘Gordon and Tina Wasson Award’ was originally established to recognize contributions from non-traditional mycologists and its name was recently changed to the ‘Community Mycologist Award’ to better align with its intended purpose and the MSA’s values. The award was established to highlight significant contributions to the field of mycology by people with non-traditional academic backgrounds. This important distinction recognizes the value of diverse perspectives in the MSA and the immensurate contributions of mycologists working outside of academia.

Gordon and Tina Wasson were non-academic mycologists, widely credited with introducing the Western world to psychoactive fungi. The decision to rename the award stemmed from concerns that its namesakes’ actions caused documented harm to indigenous communities and violated principles of research ethics. The Wasson’s work involved breaches of trust with traditional knowledge holders, including the Mazatec Curandero shaman María Sabina, who shared sacred practices under explicit conditions of confidentiality (Faudree 2013; Lutkajtis 2019). Despite clear agreements to protect sensitive cultural information, the Wassons proceeded to publish detailed accounts in popular media. These actions had complex and long-lasting effects on Sabina and the native community that entrusted the Wassons with their knowledge.

The name change acknowledges the need to address past injustices, cultural appropriation, indigenous rights, and the need for equitable practices in scientific research. This shift away from eponymous awards aligns with a broader movement in scientific societies to reconsider how to honour contributions in their fields. Recent studies have shown that awards named after individuals can perpetuate existing power structures and bias selection committees, often privileging candidates who share demographic characteristics with the namesake (Gehmlich and Krause 2024a, 2024b). Moreover, eponymous awards can exclude applicants who do not see themselves represented in these historical figures, potentially discouraging participation from underrepresented groups (Bazner et al. 2021). By focusing instead on the purpose and spirit of the recognition, the new name better serves the award’s original intent to celebrate excellence in community-based mycological work.

### ﻿MSA in 2025

**Figure 4.**
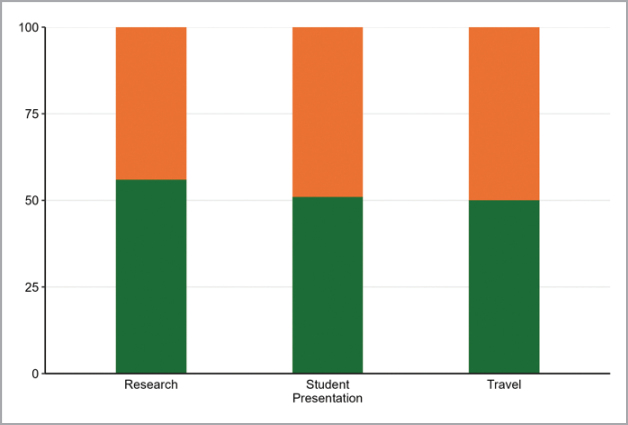
Proportion of female (orange) and male (green) recipients of MSA student awards (research, presentation, and travel) between 1969 and 2023.

**Figure 5.**
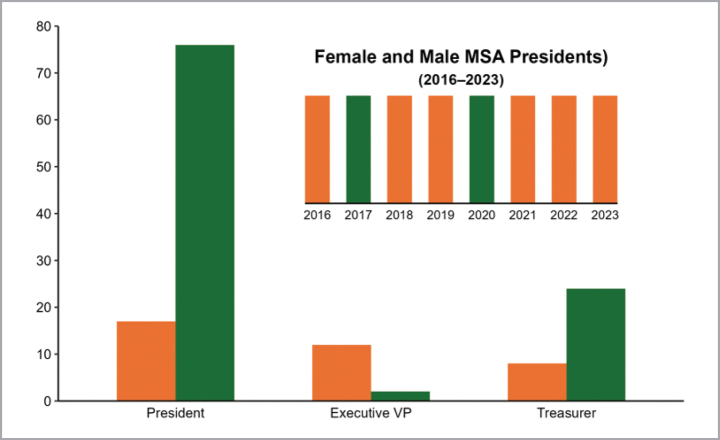
Proportion of female (orange) and male (green) MSA presidents 1932–2024 and 2016–2023 (inset).

**Figure 6.**
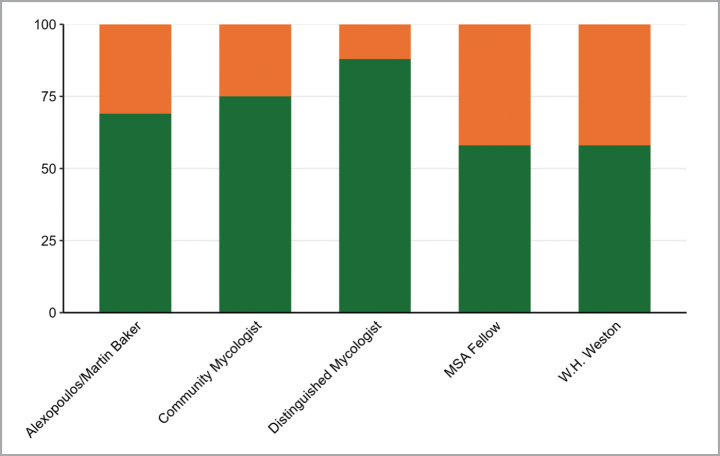
Number of male (green) and female (orange) Alexopoulos/Martin Baker (early career), Community Mycologist, Distinguished Mycologist, MSA Fellow, and Weston awardees between 1978 and 2024.

A decade of efforts to diversify MSA is slowly shifting the society towards increased inclusivity. As in 2015, the student awards are currently close to gender balanced (Fig. 4). This is a reassuring trend that we hope continues, as it accompanies the higher number of female STEM students compared to the historical record. Despite the strong historical gender imbalance documented in Branco and Vellinga (2015), in the last ten years MSA elected many female presidents (Fig. 5). There was also some progress on the professional award recipients, with close to balance in MSA Fellows and Western awardees (Fig. 6). Even though these efforts are insufficient to balance out the effects of past inequities, they are positive and an important contribution for a more diverse, equitable, and inclusive society and field.

### ﻿The next 10 years

Despite the important steps taken by the MSA over the past 10 years, there is still ample work to be done if the goal is to achieve a diverse mycology field, where equitable practices are applied by scientists that feel included and represented. Current challenges include broadening participation and representation that should be addressed in the coming years.

There are currently several demographic axes that have little representation at MSA and that would seriously contribute to achieving diverse scientific visions, enrich the mycological community, and lead to novel ideas for research, education, and outreach.

To expand membership, MSA should continue recruitment efforts, especially undergraduate students, postdoctoral researchers, and postbaccalaureates as these categories are currently underrepresented. The society should also continue to actively recruit and support students. This can be achieved through for example by continuing to support the MSA Student and Postdoc Section and the SPORES program, including expanding it to graduate students as a way to recruit new members at junior stages that can become long lasting members and assure the sustainability of MSA.

Another avenue for diversification would be for MSA to establish partnerships. These could include other mycological societies (professional and amateur, nationally and abroad), universities (including Minority Serving Institutions that serve underrepresented student bodies), scientific networks, governmental agencies, and industry. Establishing these connections could easily consolidate the current general interest in fungi while bringing together different perspectives and approaches to studying fungi that will both advance the field and promote diverse career path options.

We expect MSA will focus the next 10 years on addressing these issues and more. It will be exciting to expand efforts to promote and advance the science of mycology, as well as foster and encourage research and education in mycology in all its aspects.

## ﻿Additional information

### Data availability

All of the data that support the findings of this study are available in the main text.
